# Crystal structure of 4-(2,2-di­methyl­propanamido)­pyridin-3-yl *N*,*N*-diiso­propyl­dithio­carbamate

**DOI:** 10.1107/S1600536814019321

**Published:** 2014-08-30

**Authors:** Gamal A. El-Hiti, Keith Smith, Amany S. Hegazy, Mohammed Baashen, Benson M. Kariuki

**Affiliations:** a Cornea Research Chair, Department of Optometry, College of Applied Medical Sciences, King Saud University, PO Box 10219, Riyadh 11433, Saudi Arabia; b School of Chemistry, Cardiff University, Main Building, Park Place, Cardiff CF10 3AT, Wales; c Criminal Evidence, Ministry of Interior, Riyadh 11632, PO Box 86985, Saudi Arabia

**Keywords:** crystal structure, di­thio­carbamate, pyridine derivatives, hydrogen bonding

## Abstract

In the title compound, C_17_H_27_N_3_OS_2_, the amide group is approximately coplanar with the pyridine ring [dihedral angle = 1.6 (1)°], whereas the di­thio­carbamate group is nearly perpendicular to the pyridine ring [dihedral angle = 76.7 (1)°]. In the crystal, pairs of weak C—H⋯O hydrogen bonds link the mol­ecules into inversion dimers.

## Related literature   

For background to pyridine derivatives, see: Joule & Mills (2000 ▶); Smith *et al.* (1999 ▶). For the synthesis of the title compound, see: Smith *et al.* (1988 ▶). For spectroscopic data for this compound, see: Smith *et al.* (1994 ▶). For routes to modify the pyridine ring, see: El-Hiti (2003 ▶); Turner (1983 ▶). For crystal structures of related compounds, see: El-Hiti *et al.* (2014 ▶); Koch *et al.* (2008 ▶); Mazik & Sicking (2004 ▶).
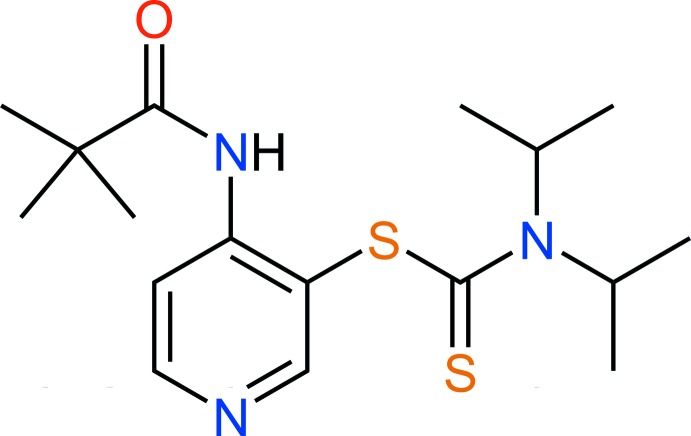


## Experimental   

### Crystal data  

C_17_H_27_N_3_OS_2_
*M* *_r_* = 353.53
Triclinic, 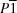

*a* = 7.9776 (7) Å
*b* = 9.5412 (9) Å
*c* = 13.0541 (14) Å
α = 83.099 (8)°
β = 83.227 (8)°
γ = 84.608 (7)°
*V* = 976.33 (17) Å^3^
*Z* = 2
Cu *K*α radiation
μ = 2.52 mm^−1^
*T* = 293 K
0.36 × 0.24 × 0.19 mm

### Data collection  

Agilent SuperNova (Dual, Cu at zero, Atlas) diffractometer
Absorption correction: multi-scan (*CrysAlis PRO*; Agilent, 2014 ▶) *T*_min_ = 0.662, *T*_max_ = 1.000
6616 measured reflections
3779 independent reflections
3391 reflections with *I* > 2σ(*I*)
*R*_int_ = 0.021

### Refinement  

*R*[*F* ^2^ > 2σ(*F* ^2^)] = 0.061
*wR*(*F* ^2^) = 0.211
*S* = 1.16
3779 reflections
215 parameters
H-atom parameters constrained
Δρ_max_ = 0.39 e Å^−3^
Δρ_min_ = −0.29 e Å^−3^

### 

Data collection: *CrysAlis PRO* (Agilent, 2014 ▶); cell refinement: *CrysAlis PRO*; data reduction: *CrysAlis PRO*; program(s) used to solve structure: *SHELXS2013* (Sheldrick, 2008 ▶); program(s) used to refine structure: *SHELXL2013* (Sheldrick, 2008 ▶); molecular graphics: *ORTEP-3 for Windows* (Farrugia, 2012 ▶); software used to prepare material for publication: *WinGX* (Farrugia, 2012 ▶).

## Supplementary Material

Crystal structure: contains datablock(s) I, New_Global_Publ_Block. DOI: 10.1107/S1600536814019321/xu5816sup1.cif

Structure factors: contains datablock(s) I. DOI: 10.1107/S1600536814019321/xu5816Isup2.hkl

Supporting information file. DOI: 10.1107/S1600536814019321/xu5816Isup3.cml

**Fig. 1.**
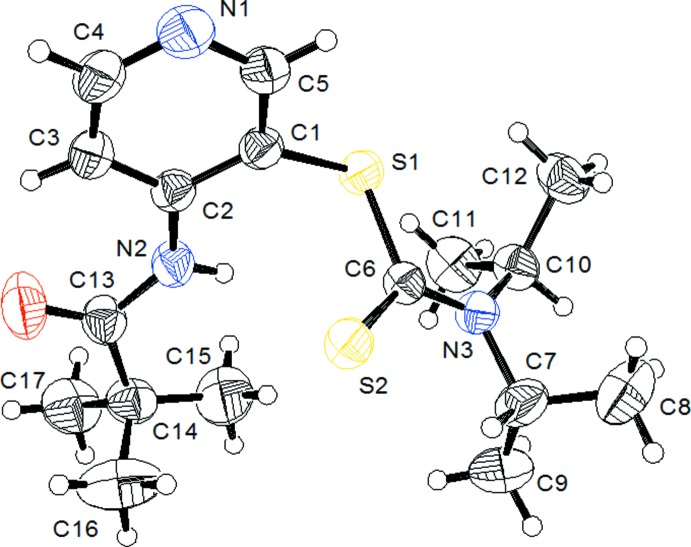
The symmetric unit of the title compound with atom labels and 50% probability displacement ellipsoids.

. DOI: 10.1107/S1600536814019321/xu5816fig1.tif
The symmetric unit of the title compound with atom labels and 50% probability displacement ellipsoids.

**Fig. 2.**
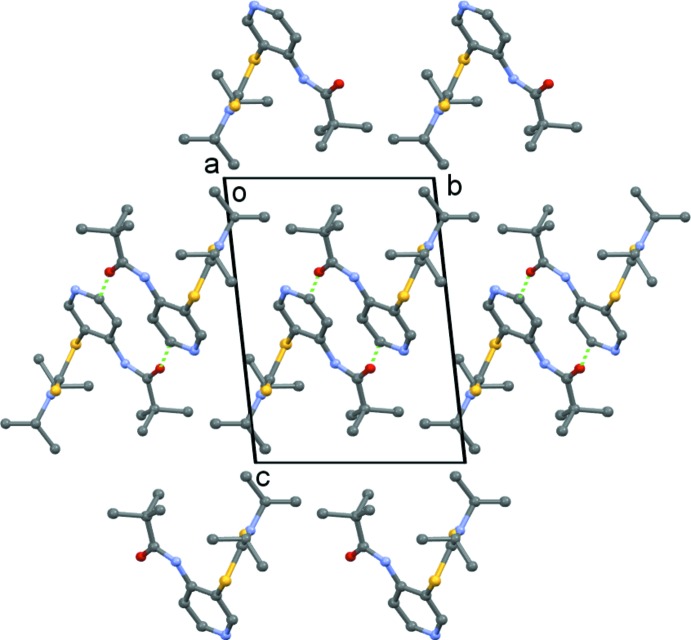
Packing in the crystal structure showing C—H···O contacts as dotted lines with hydrogen atoms omitted for clarity.

. DOI: 10.1107/S1600536814019321/xu5816fig2.tif
Packing in the crystal structure showing C—H⋯O contacts as dotted lines with hydrogen atoms omitted for clarity.

CCDC reference: 1021242

Additional supporting information:  crystallographic information; 3D view; checkCIF report

## Figures and Tables

**Table 1:** Hydrogen-bond geometry (Å, °)

*D*—H⋯*A*	*D*—H	H⋯*A*	*D*⋯*A*	*D*—H⋯*A*
C4—H4⋯O2^i^	0.93	2.54	3.447 (5)	164

Symmetry code: (i) 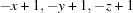.

## supplementary crystallographic information

### S1. Chemical context

Pyridine derivatives are important compounds (Joule & Mills, 2000) and various substituted derivatives can be synthesized via li­thia­tion and subsequent reaction with electrophiles (Turner, 1983). During research focused on synthesis of novel substituted pyridines (El-Hiti, 2003; Smith *et al.*, 1999) we have synthesized the title compound in high yield. For the X-ray structures for related compounds, see: El-Hiti *et al.*, 2014; Koch *et al.*, 2008; Mazik & Sicking, 2004.

### S2. Structural commentary

In the molecule of the title compound, C_17_H_27_N_3_OS_2_, (Fig. 1), the pyridine group is almost co-planar (1.6 (1)^o^) to the amide group whereas the angle to the carbamodi­thio­ate is 76.7 (1)^o^. No strong hydrogen bonding inter­actions occur, with pairs of molecules being linked by pairs of C—H..O contacts (Fig. 2). The molecular pairs are stacked along [010] leading to a structure in which the *t*-butyl groups form bilayers parallel to the *ab* plane.

### S3. Synthesis and crystallization

4-Pivalamido­pyridin-3-yl diiso­propyl­carbamodi­thio­ate was obtained in 93% yield from double li­thia­tion of 4-(pivaloyl­amino)­pyridin-3-yl with *n*-butyl­lithium at –78 to 0°C in anhydrous THF under nitro­gen followed by reaction with tetra­iso­propyl­thiuram di­sulfide (Smith *et al.*, 1988, 1994). Crystallization from ethyl acetate gave colorless crystals of the title compound. The spectroscopic data of the title compound, including NMR and low and high resolution mass spectra, were consistent with those reported (Smith *et al.*, 1994).

### S4. Refinement details

H atoms were positioned geometrically and refined using a riding model, with *U*_iso_(H) constrained to be 1.2 times *U*_eq_ for the bonded atom except for methyl groups where it was 1.5 times with free rotation about the C—C bond.

### Crystal data

**Table:**

C_17_H_27_N_3_OS_2_	*Z* = 2
*M**_r_* = 353.53	*F*(000) = 380
Triclinic, *P*1	*D*_x_ = 1.203 Mg m^−^^3^
*a* = 7.9776 (7) Å	Cu *K*α radiation, λ = 1.54184 Å
*b* = 9.5412 (9) Å	Cell parameters from 3458 reflections
*c* = 13.0541 (14) Å	θ = 4.7–73.3°
α = 83.099 (8)°	µ = 2.52 mm^−^^1^
β = 83.227 (8)°	*T* = 293 K
γ = 84.608 (7)°	Block, colourless
*V* = 976.33 (17) Å^3^	0.36 × 0.24 × 0.19 mm

### Data collection

**Table:**

Agilent SuperNova (Dual, Cu at zero, Atlas) diffractometer	3391 reflections with *I* > 2σ(*I*)
Radiation source: sealed X-ray tube	*R*_int_ = 0.021
ω scans	θ_max_ = 73.5°, θ_min_ = 4.7°
Absorption correction: multi-scan (*CrysAlis PRO*; Agilent, 2014)	*h* = −9→9
*T*_min_ = 0.662, *T*_max_ = 1.000	*k* = −11→11
6616 measured reflections	*l* = −11→16
3779 independent reflections	

### Refinement

**Table:**

Refinement on *F*^2^	0 restraints
Least-squares matrix: full	Hydrogen site location: inferred from neighbouring sites
*R*[*F*^2^ > 2σ(*F*^2^)] = 0.061	H-atom parameters constrained
*wR*(*F*^2^) = 0.211	*w* = 1/[σ^2^(*F*_o_^2^) + (0.1014*P*)^2^ + 0.897*P*] where *P* = (*F*_o_^2^ + 2*F*_c_^2^)/3
*S* = 1.16	(Δ/σ)_max_ < 0.001
3779 reflections	Δρ_max_ = 0.39 e Å^−^^3^
215 parameters	Δρ_min_ = −0.29 e Å^−^^3^

### Special details

**Table:**

Geometry. All e.s.d.'s (except the e.s.d. in the dihedral angle between two l.s. planes) are estimated using the full covariance matrix. The cell e.s.d.'s are taken into account individually in the estimation of e.s.d.'s in distances, angles and torsion angles; correlations between e.s.d.'s in cell parameters are only used when they are defined by crystal symmetry. An approximate (isotropic) treatment of cell e.s.d.'s is used for estimating e.s.d.'s involving l.s. planes.

### Fractional atomic coordinates and isotropic or equivalent isotropic displacement parameters (Å^2^)

**Table:**

	*x*	*y*	*z*	*U*_iso_*/*U*_eq_	
C1	0.0193 (4)	0.7422 (4)	0.4668 (2)	0.0459 (7)	
C2	0.1318 (4)	0.6291 (3)	0.4352 (2)	0.0452 (7)	
C3	0.2680 (5)	0.5875 (4)	0.4929 (3)	0.0537 (8)	
H3	0.3449	0.5121	0.4759	0.064*	
C4	0.2866 (5)	0.6596 (5)	0.5752 (3)	0.0635 (10)	
H4	0.3792	0.6311	0.6120	0.076*	
C5	0.0505 (5)	0.8057 (4)	0.5520 (3)	0.0565 (8)	
H5	−0.0253	0.8798	0.5727	0.068*	
C6	−0.1194 (4)	0.8903 (3)	0.2919 (2)	0.0419 (6)	
C7	−0.2165 (5)	1.0250 (5)	0.1370 (3)	0.0658 (10)	
H7	−0.0963	1.0426	0.1293	0.079*	
C8	−0.3150 (9)	1.1680 (6)	0.1370 (5)	0.0960 (17)	
H8A	−0.4341	1.1560	0.1441	0.144*	
H8B	−0.2847	1.2250	0.0729	0.144*	
H8C	−0.2889	1.2141	0.1939	0.144*	
C9	−0.2421 (9)	0.9459 (7)	0.0484 (4)	0.1004 (18)	
H9A	−0.1624	0.8641	0.0467	0.151*	
H9B	−0.2250	1.0063	−0.0156	0.151*	
H9C	−0.3552	0.9165	0.0573	0.151*	
C10	−0.4303 (4)	0.9068 (4)	0.2679 (3)	0.0534 (8)	
H10	−0.4906	0.9553	0.2108	0.064*	
C11	−0.4589 (6)	0.7522 (5)	0.2694 (4)	0.0733 (12)	
H11A	−0.4032	0.7174	0.2071	0.110*	
H11B	−0.5781	0.7418	0.2736	0.110*	
H11C	−0.4135	0.6990	0.3285	0.110*	
C12	−0.5115 (5)	0.9746 (5)	0.3641 (3)	0.0704 (11)	
H12A	−0.4714	0.9220	0.4250	0.106*	
H12B	−0.6324	0.9739	0.3684	0.106*	
H12C	−0.4816	1.0705	0.3592	0.106*	
C13	0.2026 (5)	0.4709 (4)	0.2956 (3)	0.0554 (8)	
C14	0.1560 (6)	0.4522 (4)	0.1882 (3)	0.0613 (9)	
C15	−0.0198 (8)	0.5172 (7)	0.1668 (4)	0.0987 (18)	
H15A	−0.1028	0.4767	0.2183	0.148*	
H15B	−0.0419	0.4983	0.0992	0.148*	
H15C	−0.0255	0.6177	0.1693	0.148*	
C16	0.2920 (10)	0.5217 (8)	0.1114 (4)	0.113 (2)	
H16A	0.2806	0.5005	0.0427	0.169*	
H16B	0.4020	0.4859	0.1301	0.169*	
H16C	0.2785	0.6225	0.1134	0.169*	
C17	0.1623 (8)	0.2942 (5)	0.1788 (4)	0.0875 (15)	
H17A	0.0784	0.2520	0.2289	0.131*	
H17B	0.2725	0.2507	0.1914	0.131*	
H17C	0.1399	0.2803	0.1102	0.131*	
N1	0.1823 (5)	0.7673 (4)	0.6064 (3)	0.0678 (9)	
N2	0.1026 (4)	0.5683 (3)	0.3481 (2)	0.0523 (7)	
H2	0.0079	0.5961	0.3239	0.063*	
N3	−0.2495 (3)	0.9371 (3)	0.2384 (2)	0.0467 (6)	
O2	0.3244 (5)	0.4055 (4)	0.3306 (3)	0.0946 (12)	
S1	−0.17473 (10)	0.79230 (10)	0.41574 (6)	0.0518 (3)	
S2	0.08414 (10)	0.91374 (10)	0.25296 (7)	0.0535 (3)	

### Atomic displacement parameters (Å^2^)

**Table:**

	*U*^11^	*U*^22^	*U*^33^	*U*^12^	*U*^13^	*U*^23^
C1	0.0432 (16)	0.0523 (17)	0.0417 (15)	−0.0037 (13)	−0.0063 (12)	−0.0018 (13)
C2	0.0450 (17)	0.0487 (17)	0.0425 (15)	−0.0054 (13)	−0.0070 (13)	−0.0038 (12)
C3	0.0479 (19)	0.061 (2)	0.0522 (18)	0.0012 (15)	−0.0112 (15)	−0.0045 (15)
C4	0.055 (2)	0.083 (3)	0.055 (2)	−0.0003 (19)	−0.0214 (17)	−0.0078 (18)
C5	0.058 (2)	0.064 (2)	0.0489 (18)	0.0016 (16)	−0.0091 (15)	−0.0113 (15)
C6	0.0387 (15)	0.0442 (15)	0.0439 (15)	−0.0038 (12)	−0.0051 (12)	−0.0079 (12)
C7	0.053 (2)	0.087 (3)	0.055 (2)	−0.0067 (19)	−0.0119 (16)	0.0123 (19)
C8	0.117 (5)	0.075 (3)	0.093 (4)	−0.006 (3)	−0.029 (3)	0.017 (3)
C9	0.125 (5)	0.124 (5)	0.048 (2)	0.006 (4)	−0.005 (3)	−0.007 (3)
C10	0.0340 (16)	0.072 (2)	0.0564 (19)	−0.0040 (15)	−0.0084 (14)	−0.0128 (16)
C11	0.054 (2)	0.082 (3)	0.090 (3)	−0.021 (2)	−0.011 (2)	−0.019 (2)
C12	0.046 (2)	0.097 (3)	0.070 (2)	0.007 (2)	−0.0047 (17)	−0.025 (2)
C13	0.056 (2)	0.0511 (18)	0.061 (2)	0.0001 (15)	−0.0111 (16)	−0.0131 (15)
C14	0.072 (2)	0.059 (2)	0.055 (2)	−0.0088 (18)	−0.0056 (18)	−0.0155 (16)
C15	0.111 (4)	0.120 (4)	0.076 (3)	0.014 (3)	−0.046 (3)	−0.040 (3)
C16	0.142 (6)	0.136 (5)	0.066 (3)	−0.069 (5)	0.002 (3)	−0.005 (3)
C17	0.111 (4)	0.071 (3)	0.087 (3)	−0.011 (3)	−0.009 (3)	−0.033 (2)
N1	0.068 (2)	0.084 (2)	0.0559 (18)	−0.0008 (18)	−0.0189 (16)	−0.0206 (16)
N2	0.0496 (16)	0.0579 (16)	0.0520 (15)	0.0043 (13)	−0.0158 (12)	−0.0134 (13)
N3	0.0377 (13)	0.0586 (16)	0.0447 (14)	−0.0029 (11)	−0.0089 (11)	−0.0055 (11)
O2	0.090 (2)	0.098 (2)	0.102 (2)	0.042 (2)	−0.041 (2)	−0.045 (2)
S1	0.0388 (4)	0.0662 (5)	0.0473 (5)	−0.0015 (3)	−0.0035 (3)	0.0026 (4)
S2	0.0376 (4)	0.0614 (5)	0.0600 (5)	−0.0087 (3)	−0.0049 (3)	0.0029 (4)

### Geometric parameters (Å, º)

**Table:**

C1—C5	1.386 (5)	C10—C11	1.511 (6)
C1—C2	1.406 (5)	C10—C12	1.530 (5)
C1—S1	1.760 (3)	C10—H10	0.9800
C2—N2	1.390 (4)	C11—H11A	0.9600
C2—C3	1.395 (5)	C11—H11B	0.9600
C3—C4	1.373 (5)	C11—H11C	0.9600
C3—H3	0.9300	C12—H12A	0.9600
C4—N1	1.332 (5)	C12—H12B	0.9600
C4—H4	0.9300	C12—H12C	0.9600
C5—N1	1.336 (5)	C13—O2	1.210 (5)
C5—H5	0.9300	C13—N2	1.361 (5)
C6—N3	1.331 (4)	C13—C14	1.527 (5)
C6—S2	1.671 (3)	C14—C15	1.522 (7)
C6—S1	1.797 (3)	C14—C17	1.523 (6)
C7—N3	1.489 (5)	C14—C16	1.530 (7)
C7—C9	1.498 (7)	C15—H15A	0.9600
C7—C8	1.509 (7)	C15—H15B	0.9600
C7—H7	0.9800	C15—H15C	0.9600
C8—H8A	0.9600	C16—H16A	0.9600
C8—H8B	0.9600	C16—H16B	0.9600
C8—H8C	0.9600	C16—H16C	0.9600
C9—H9A	0.9600	C17—H17A	0.9600
C9—H9B	0.9600	C17—H17B	0.9600
C9—H9C	0.9600	C17—H17C	0.9600
C10—N3	1.494 (4)	N2—H2	0.8600
			
C5—C1—C2	118.5 (3)	C10—C11—H11C	109.5
C5—C1—S1	117.4 (3)	H11A—C11—H11C	109.5
C2—C1—S1	123.5 (2)	H11B—C11—H11C	109.5
N2—C2—C3	124.4 (3)	C10—C12—H12A	109.5
N2—C2—C1	118.3 (3)	C10—C12—H12B	109.5
C3—C2—C1	117.3 (3)	H12A—C12—H12B	109.5
C4—C3—C2	118.8 (3)	C10—C12—H12C	109.5
C4—C3—H3	120.6	H12A—C12—H12C	109.5
C2—C3—H3	120.6	H12B—C12—H12C	109.5
N1—C4—C3	125.0 (3)	O2—C13—N2	122.1 (4)
N1—C4—H4	117.5	O2—C13—C14	121.6 (4)
C3—C4—H4	117.5	N2—C13—C14	116.2 (3)
N1—C5—C1	124.3 (4)	C15—C14—C17	107.8 (4)
N1—C5—H5	117.9	C15—C14—C13	114.1 (3)
C1—C5—H5	117.9	C17—C14—C13	108.3 (4)
N3—C6—S2	125.8 (2)	C15—C14—C16	110.6 (5)
N3—C6—S1	115.0 (2)	C17—C14—C16	110.7 (4)
S2—C6—S1	119.17 (18)	C13—C14—C16	105.3 (4)
N3—C7—C9	111.2 (4)	C14—C15—H15A	109.5
N3—C7—C8	111.3 (4)	C14—C15—H15B	109.5
C9—C7—C8	114.1 (4)	H15A—C15—H15B	109.5
N3—C7—H7	106.6	C14—C15—H15C	109.5
C9—C7—H7	106.6	H15A—C15—H15C	109.5
C8—C7—H7	106.6	H15B—C15—H15C	109.5
C7—C8—H8A	109.5	C14—C16—H16A	109.5
C7—C8—H8B	109.5	C14—C16—H16B	109.5
H8A—C8—H8B	109.5	H16A—C16—H16B	109.5
C7—C8—H8C	109.5	C14—C16—H16C	109.5
H8A—C8—H8C	109.5	H16A—C16—H16C	109.5
H8B—C8—H8C	109.5	H16B—C16—H16C	109.5
C7—C9—H9A	109.5	C14—C17—H17A	109.5
C7—C9—H9B	109.5	C14—C17—H17B	109.5
H9A—C9—H9B	109.5	H17A—C17—H17B	109.5
C7—C9—H9C	109.5	C14—C17—H17C	109.5
H9A—C9—H9C	109.5	H17A—C17—H17C	109.5
H9B—C9—H9C	109.5	H17B—C17—H17C	109.5
N3—C10—C11	113.1 (3)	C4—N1—C5	116.0 (3)
N3—C10—C12	113.3 (3)	C13—N2—C2	129.2 (3)
C11—C10—C12	114.6 (4)	C13—N2—H2	115.4
N3—C10—H10	104.9	C2—N2—H2	115.4
C11—C10—H10	104.9	C6—N3—C7	118.8 (3)
C12—C10—H10	104.9	C6—N3—C10	126.5 (3)
C10—C11—H11A	109.5	C7—N3—C10	114.7 (3)
C10—C11—H11B	109.5	C1—S1—C6	104.98 (15)
H11A—C11—H11B	109.5		

### Hydrogen-bond geometry (Å, º)

**Table:**

*D*—H···*A*	*D*—H	H···*A*	*D*···*A*	*D*—H···*A*
C4—H4···O2^i^	0.93	2.54	3.447 (5)	164

Symmetry code: (i) −*x*+1, −*y*+1, −*z*+1.
