# Supplementary figures and images for: Crystal structure of 4-(2,2-di­methyl­propanamido)­pyridin-3-yl N,N-diiso­propyl­dithio­carbamate
Source: Acta Crystallogr Sect E Struct Rep Online. 2014 Aug 30;70(Pt 9):o1069–70. doi: 10.1107/S1600536814019321 (PMC4186077; doi:10.1107/S1600536814019321)

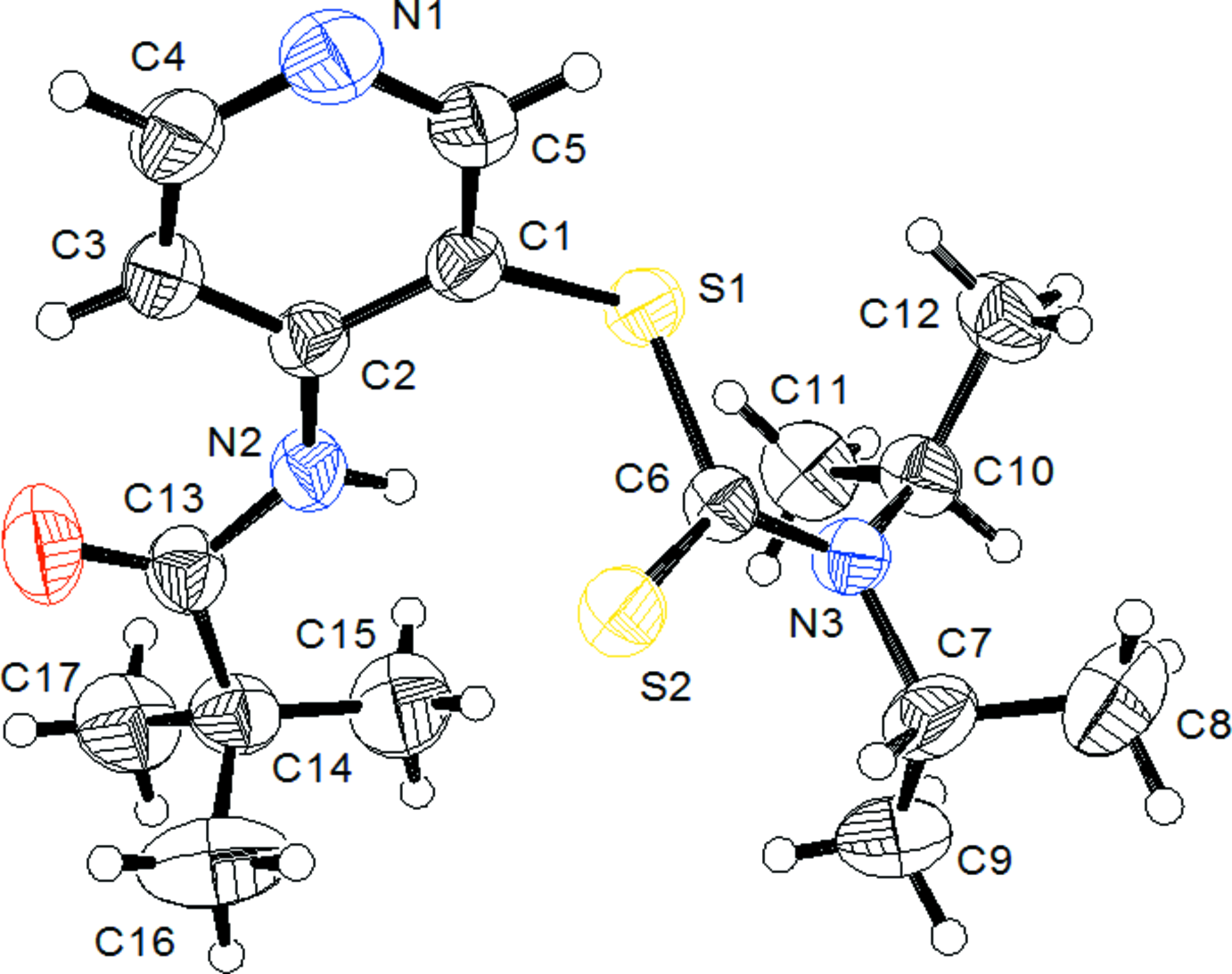

Supplement: Supplementary file 4 [file e-70-o1069-fig1.tif]

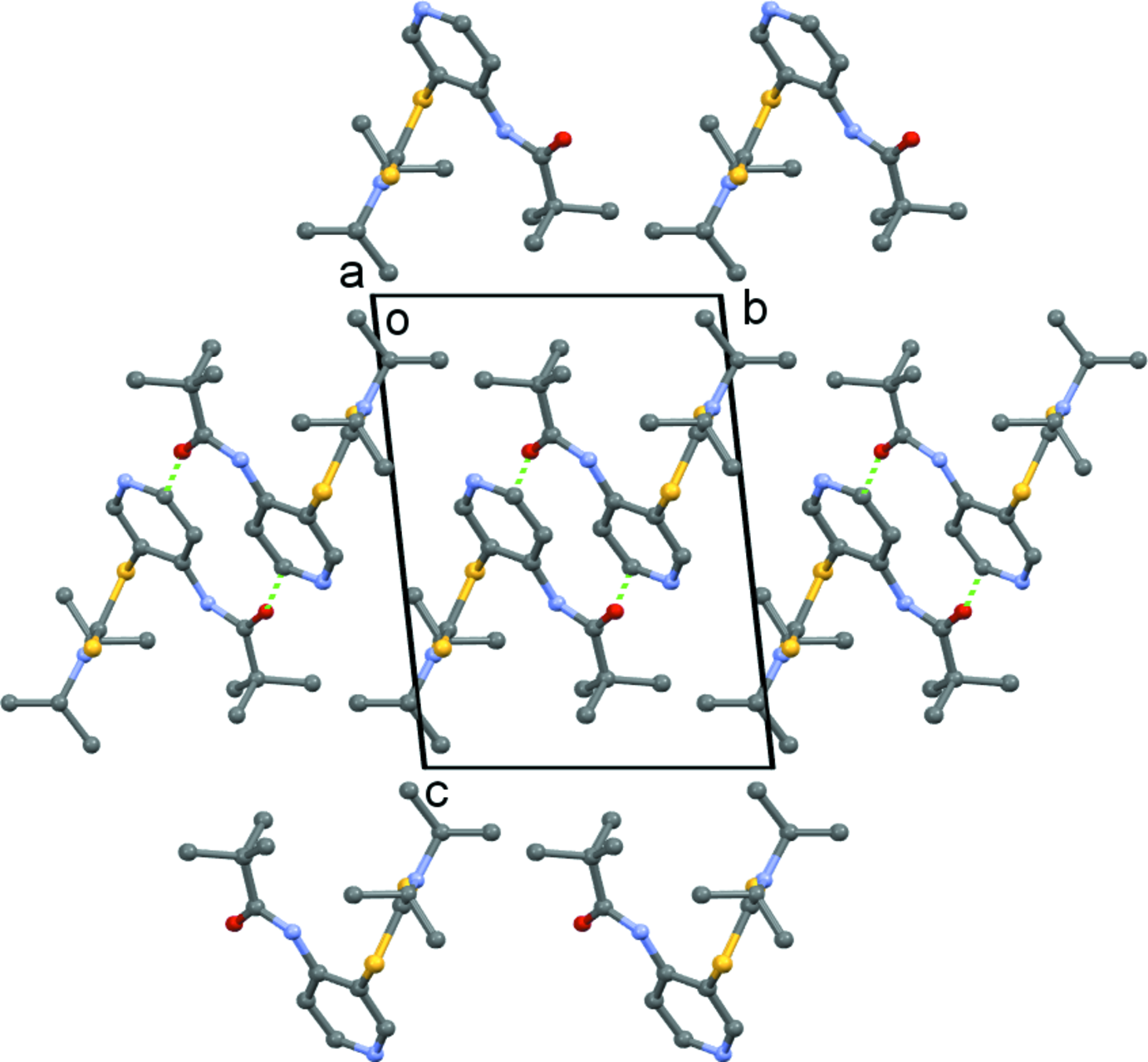

Supplement: Supplementary file 5 [file e-70-o1069-fig2.tif]
